# The unseen epidemic: trauma and loneliness in urban midlife women

**DOI:** 10.1186/s40695-022-00080-z

**Published:** 2022-10-27

**Authors:** E Liberatore-Maguire, A Devlin, S Fisher, F Ramsey, H Grunwald, K Brownstein, MF Morrison

**Affiliations:** 1 Temple University Health System, Department of Psychiatry, Temple University Hospital, Episcopal Campus, 100 E. Lehigh Avenue MAB Suite 305B, Philadelphia, PA 19125 USA; 2 Lewis Katz School of Medicine, Department of Clinical Sciences, Temple University, 3440 North Broad Street, Kresge East Room 211, Philadelphia, PA 19140 USA; 3 Dornsife School of Public Health, Drexel University, Philadelphia, PA 19104 USA; 4 Public Health Law Research, Beasley School of Law, 1819 North Broad Street, Barrack Hall, Suite 300, Philadelphia, PA 19122 USA

**Keywords:** Loneliness, Trauma, Midlife, Female, Minority, Mental health, Women

## Abstract

**Background:**

Connectedness and attachment are vital parts of humanity. Loneliness, a state of distress in reaction to perceived detachment and isolation, is reported by over one-third of U.S. adults and is associated with numerous physical and mental health consequences. What contributes to loneliness, especially in women and minority populations, is poorly understood, but this population is also at greater risk for abuse and trauma. Our study aimed to further understand loneliness in urban midlife women and to explore the relationship that may exist with trauma(s).

**Methods:**

To identify primacies for mental health care, female midlife participants (*N=*50) of a long-standing urban community-based cohort focused on health improvement completed a one-time audiotaped interview with both quantitative assessments and a qualitative interview. Loneliness was assessed by the UCLA 3-item Loneliness Scale. Using semi-structured interviews, open-ended questions facilitated a discussion regarding mental health needs and experiences. Interview transcripts were coded and analyzed following a grounded theory methodology. Themes around loneliness and trauma emerged. The transcripts were coded using the same methodology and coders as the individual interviews. Twenty women participated in two optional focus groups.

**Results:**

Participants had a mean age of 50, with the majority identifying as Black/African American (*N=*37) and unemployed (*N=*33). Three themes emerged regarding perceived causes of loneliness: trauma, the burden of responsibilities for others, and secondary to unhealthy relationships. Loneliness associated with trauma will be explored here; other themes are beyond the scope of this paper and will be discussed in subsequent analyses. Quantitative results suggest that physical abuse (loneliness scores 5.4 vs. 4.0, *p=*0.003), as well as emotional abuse and neglect (loneliness scores 5.6 vs. 4.4, *p=*0.01), were associated with greater loneliness.

**Conclusion:**

In urban midlife low-income women, lifetime physical abuse and emotional abuse/neglect are associated with increased feelings of loneliness. Qualitative data provide insight into how participants viewed their traumatic histories, ways in which the trauma has ongoing influence, and how they experience loneliness. Though further investigation is needed, trauma-informed approaches should be considered in both primary care and mental health settings with a focus on mitigating loneliness and providing appropriate support and trauma treatment.

**Supplementary Information:**

The online version contains supplementary material available at 10.1186/s40695-022-00080-z.

## Background

The need to feel connected is profoundly human. From birth we are wired to seek attachment and comfort in relationships [44]. It is through relatedness—through establishing, nurturing, and preserving relationships—that we satisfy our innate need to belong, to see and be seen. Loneliness is a state of distress in reaction to perceived isolation or disconnectedness. It is a subjective experience independent of the actual state of being alone [38]. For most, it is an experience of deprivation, with a sobering risk for abject consequences.

During infancy, connectedness with a caregiver not only ensures physical survival, but is essential to the psychological and social development that eventually allows for a separate and secure existence. In the 1940s, Bawkin and Spitz both wrote on the devastating effects of psychological neglect and caregiver deprivation—institutionalized children, void of human contact and emotional connection, had higher death rates [1, 45]. The lethality of loneliness was realized [35] and the primacy of relationships to normal human development would become the basis of Bowlby’s and Ainsworth’s attachment theories [4].

As advances in neuroscience have been made, we have also recognized the biological and neurodevelopmental role of nurturing connections [37]. Early relationship disruptions, such as neglect—arguably an extreme of loneliness—have been associated with structural and functional abnormalities in brain regions involved in regulation of stress and affect [19] and the hypothalamic-pituitary-adrenal axis [5].

Loneliness has a detrimental impact on mental health, with links to suicidality—both suicidal ideation and attempts [49]—and there is a growing body of evidence to suggest that the painful experience of disconnectedness begins with an overlap in the neural circuitry responsible for both physical and social pain [16]. Multiple studies have shown loneliness is associated with an increased risk for depression [6, 57] and anxiety [3, 28], it has also been linked to overall poorer mental health outcomes [56] and psychosis [7].

The physical health consequences of loneliness have been equated to smoking 15 cigarettes per day [23]. Loneliness has been associated with hypertension [22], poor immune response [39], coronary artery disease [2, 54], negative physiologic responses to stress and poor sleep [48], dementia [24], and cognitive decline [15]. In a 2015 meta-analysis of prospective studies that totaled 3.4 million participants followed for an average of seven years, loneliness was associated with a 26% increased likelihood of mortality [47].

Both the mental and physical health implications of loneliness are apparent. With recent surveys in the U.S. finding that 22-35% of adults experience loneliness, the burden of this malady is significant [14, 52]. The potential individual and societal ramifications have motivated efforts to identify both what may be contributing to loneliness as well as possible interventions.

Studies have found that social disadvantage [53], female gender [3], and ethnic minority status [31] are all associated with greater loneliness. Despite these findings, further research on what contributes to loneliness in these populations is lacking [12], with many studies identifying the risk factors for loneliness having been conducted in older adults and populations outside of the US [50].

Research into the determinants of loneliness has found that both trauma [9] and risky childhood family environments [10] were predictors of loneliness, though these studies were conducted in an older Israeli and college undergraduate population, respectively. Additionally, national US surveys discovered that half of adult women reported exposure to a traumatic event at some point in their life [18]. The socially disadvantaged [33] along with racial and ethnic minorities are especially vulnerable, with 61% of Black and 51% of Hispanic children having at least one Adverse Childhood Experience [42].

The Temple University Hospital System (TUHS) community health needs assessment identified loneliness as a mental health priority in 2013 [51]. TUH serves North Philadelphia. The community is young, with a median age of 32, and home to a substantial minority population (46% Black and 30% Hispanic). Over half (53%) of the residents are women, frequently living with significant social challenges and deep poverty [26]. In exploring loneliness in this population, we utilized semi-structured interviews and focus groups to allow themes to emerge, in addition to using quantitative scales in the individual interviews. Utilizing grounded theory methodology, this study aims to expand on current research by looking at loneliness in socially disadvantaged, mostly minority midlife women and further exploring the potential link to trauma. The interviews shed a unique light on the experiences of these women and help increase understanding of the connection between feelings of loneliness and traumatic events in their lives.

## Methods

### Study population

Study participants were recruited between February 2017 and April 2018. Eligible individuals were 35-60 years old, fluent in English, identified as women, and were enrolled in Temple Health: Block-by-Block (THB3), a community-based longitudinal cohort study out of Temple University. THB3 enrolled participants living in the 11 ZIP codes that comprise Temple University Hospital’s North Philadelphia catchment area, an economically distressed urban neighborhood. The THB3 study aimed to enroll a study population reflecting the surrounding neighborhoods and was designed to interface directly with community members. The goal was to develop a sustainable cohort of residents engaged in individual and community health improvement through health research. Study participants were recruited via door-to-door canvassing and community events, and were asked to complete semi-annual surveys on a variety of health and behavioral topics. Complete methods for THB3 have been published elsewhere [17]. Women from the cohort meeting the eligibility criteria were contacted by research staff to ask whether they would be interested in completing a one-time individual interview to explore women’s mental health needs and experiences. Upon completion of the interview, participants were also invited to take part in an optional focus group.

### Interview and interview guide

Individual interviews were semi-structured. The interview guide included a variety of quantitative scales, both validated and investigator-derived, which supplemented demographic data collected during routine THB3 study visits. Topics covered included anxiety, depression, loneliness, and trauma, as described below.

To assess loneliness, we utilized the 3-item UCLA Loneliness Scale (UCLA-3) [27]. Loneliness questions were gauged with the 4-item response selection found in the 20-item UCLA Loneliness Scale [41] and converted to a 3-item scale to make data comparable to other studies. More information can be found in Additional file 1: Appendix A. Anxiety was assessed using the Generalized Anxiety Disorder 7-item (GAD-7) scale [46]. Depression was assessed with the Patient Health Questionnaire 9 (PHQ-9) [30]. We developed a Trauma Scale (TS) with questions adapted from the Trauma History Questionnaire (THQ) [25] and the Stressful Life Events Screening Questionnaire (SLESQ) [21] to assess exposure to traumatic events. These traumatic events included childhood and lifetime physical abuse/assault, neglect, sexual and emotional abuse, and having witnessed violence. We developed an additional question (question 8), which asked about living without a biological parent prior to age 18. This question was added in response to four pilot interviews suggesting that some of our study population had lived without their parents, having been placed with relatives, in the foster system, or in “therapeutic” schools. As not all assessed potentially traumatic events were specifically types of abuse (e.g., witnessing violence), trauma is used as the larger, more inclusive term. A Total Trauma Score was calculated with one point being given for every affirmative answer to a categorical yes or no question about types of abuse/trauma experienced. A score was also given for number of abuse events experienced in each category and cumulatively (Total Trauma Events). Full adaptation and scoring information can be found in Additional file 1: Appendix B.

In addition to these quantitative scales, participants completed a qualitative interview that covered a variety of domains surrounding mental health. During the interview and optional focus group, participants were asked to expand on their experiences and the associated effects on their daily lives. Themes derived from the individual interviews informed covered topics in the two follow-up focus groups, which were attended by 20 women in total. No quantitative data were derived from these focus groups.

The interview guide and study documents were reviewed and approved by the Temple University IRB and subjects received a $40 gift card for their time, the usual compensation for a long interview. Written informed consent was obtained from all participants.

### Data collection

All data, quantitative and qualitative, were collected via verbal interview. The majority of interviews were conducted in a private area of the woman’s home, though some were completed in research offices to accommodate participant requests. Two (of four) female field specialists, with master’s degrees and diverse ethnicity and backgrounds, conducted all interviews to ensure consistency. One field specialist served as the primary interviewer and the other as notetaker. Interviewers often had previously established relationships with participants through the THB3 parent study.

According to participant consent, interviews were audio recorded and recordings were transcribed upon interview completion and reviewed by another member of the study team to ensure accuracy. Three participants declined to be audio recorded. For these non-recorded interviews, detailed notes were taken to capture interview content. Interviews ranged from 25 to 215 minutes (mean length of time=75mins).

### Transcript analysis

Qualitative data were analyzed in accordance with grounded theory. All transcripts were stripped of identifiers and then coded by pairs of researchers. The pairs analyzed the transcripts line-by-line. Coding was done as a group using a hierarchical coding process [40], which allowed coders to discuss themes as they arose. Themes were developed from the data. A rudimentary coding guide was then developed and revised as data were collected [11]. The coding guide for themes included a definition, usage instructions, and examples of use [13]. After each pair coded five transcripts, the pairs were rearranged to prevent coding drift. To ensure coding consistency, group coding meetings were held routinely. The purpose of these meetings was to ensure consistent use of the codes, as well as to resolve any disagreements within the coding pairs. Ten percent of coded interviews were reviewed at a group coding meeting. A total of 5 staff members coded the interviews, four of whom conducted interviews. All transcripts were coded, and coding was done using NVivo 11 Software.

### Statistical approach

Specific quantitative data instruments have been previously described. In analyzing the quantitative data, the 3-item UCLA loneliness total score was treated as a continuous variable. Based on normality testing, the data were sufficiently normally distributed to support the application of parametric methods for analyses. Further analytical focus was to identify factors having significant associations with outcomes of interest (e.g., the 3-item UCLA Loneliness Scale). Specific statistical analysis techniques depended on the nature of the variables being analyzed. Data describing a categorial group and a continuous outcome were assessed using either the two-sample t-test or ANOVA, and the association of two continuous variables was assessed using Pearson’s correlation coefficient.

Statistical significance was defined as *p*<0.05. All reported *p*-values are two-sided where applicable. Reflective of the exploratory nature of this research, multiple testing adjustments (e.g., Bonferroni correction) were not applied. Statistical analyses were conducted using SAS® 9.4 (SAS Institute, Cary, NC).

## Results

### Demographics

Women (*N=*50) in this study had a mean age of 50.2 ± 8.6 years. Demographic information can be found in Table 1. The majority of the sample were African American non-Hispanic (74%), single (50%), and unemployed (66%). Seventy percent self-identified as having experienced previous mental health struggles—this was not defined by investigators and did not require diagnosis by a mental health professional. At the time of the assessment, 78% screened positive for anxiety and 64% for depression with mean scores on the GAD-7 and PHQ-9 of 10.8 and 12.4 respectively (moderate severity).

**Table 1:** Characteristics of the women (*N=*50)

**Age (years)**		
Mean ± SD	50.2 ± 8.6	
Range	35-60	
**Race**		
African American non-Hispanic	74%	37
Hispanic	20%	10
White non-Hispanic	4%	2
Other	2%	1
**Partner status**		
Single	50%	25
Partnered	18%	9
Married	20%	10
Divorced, widowed, other	12%	6
**Employment status**		
Employed	34%	17
Not employed	66%	33
**Level of education**		
Did not complete high school/equivalent	12%	6
High school graduate	88%	44
**Previous mental health struggles**		
Yes	70%	35
No	30%	15
Reported anxiety	78%	39
GAD-7 mean score	10.8	
Reported depression	64%	32
PHQ-9 mean score	12.4	

### The experience of loneliness

**Fig. 1:**
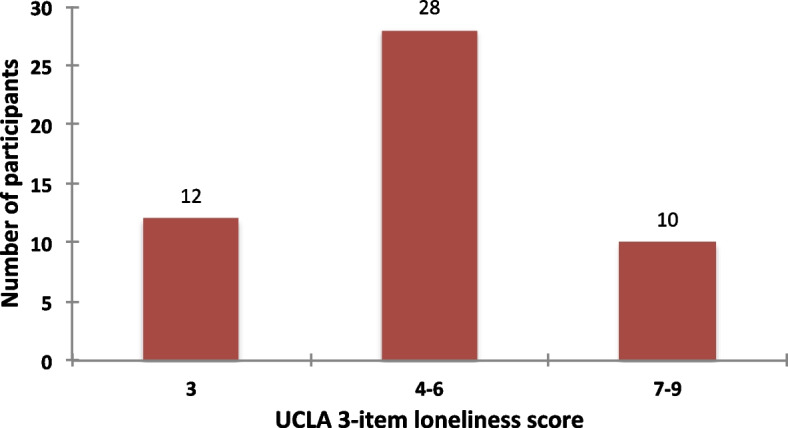
Total loneliness scores

A great majority of our participants (76%) reported some degree of feeling alone with 20% experiencing significant loneliness (loneliness score ≥ 7) (Fig. 1). During the semi-structured interviews and focus groups, the participants were given the space to share their experiences, thoughts, and feelings. Loneliness was described as “*a need for companionship... somebody to talk to,”* and feeling *“desolate, empty... detached.”*

Many women identified the way social isolation factors in with descriptions of lacking companionship and feeling “*like I’m in this world all by myself.”* Others spoke of the loneliness that exists despite having others around:

*“I don’t feel isolation, like, physically, but... mentally—where I’m at and what I’m going through... I just feel like, you know, some stuff I just can’t talk to nobody about because I feel like they’re not going to understand.”*

One woman described the persistent feeling of loneliness throughout her life despite being surrounded by others: *“Mentally I grew up alone, even though I was in a house, a family, and people around me... Mentally, I was broken.”* Another conveyed the disconnection that comes from feeling that others don’t share in your experience*: “The other type of loneliness is just feeling like maybe people don't really understand, you know?”*

### Loneliness themes

In addition to the descriptions of loneliness, three themes emerged from the interviews and focus groups in regard to the perceived causes of loneliness: loneliness associated with trauma, loneliness due to the burden of responsibilities for others, and loneliness secondary to unhealthy relationships. For this paper, the focus will be on the experience of trauma and how that relates to loneliness.

### Analyses of trauma type

Trauma was common in the study population, with all but three participants reporting at least one traumatic event in their life. Eighty percent recounted a history of physical abuse, 58% of sexual abuse, and 62% of emotional abuse or neglect (Table 2). Just over half (52%) had witnessed violence and/or been raised by non-biological guardians. Of these, both physical and emotional abuse were found to be significantly associated with loneliness, while no association was seen with sexual abuse, witnessing violence, or being raised by non-biological guardians.

**Table 2:** UCLA 3-Item Loneliness Scale by type of abuse (categorical independent variables), t-test

	N	MLS	SD	95% CI	p
Physical Abuse					0.003
Yes	40	5.4	1.9	[4.8, 6]	
No	10	4.0	0.9	[3.3, 4.7]	
Sexual Abuse					0.94
Yes	29	5.1	1.8	[4.4, 5.8]	
No	21	5.1	2	[4.2, 6]	
Emotional Abuse or Neglect					0.01
Yes	31	5.6	2	[4.8, 6.3]	
No	19	4.4	1.2	[3.8, 4.9]	
Witnessed Violence					0.06
Yes	26	4.7	1.7	[4, 5.3]	
No	24	5.6	1.9	[4.8, 6.4]	
Lived with non-bio parent					0.29
Yes	26	5.4	2.1	[4.5, 6.2]	
No	24	4.8	1.4	[4.2, 5.4]	

*MLS* Mean Loneliness Score

*p*-value based on student’s t-test

### Analyses of cumulative trauma exposure and loneliness

Given the high prevalence of trauma in our population, on an exploratory basis, we also looked at the association of loneliness with cumulative trauma events to probe whether a relationship was present. Cumulative trauma was examined from both an event (Total Trauma Events) and type (Total Trauma Score) count (Table 3). Higher trauma event counts were associated with higher loneliness scores (r=0.440, p=0.001). Analysis of the cumulative effect of experiencing different types of trauma showed a weak correlation (r=0.26), which did not meet statistical significance (*p*=0.064).

**Table 3:** UCLA 3-Item Loneliness Scale by trauma event and type counts (continuous independent variables), Pearson’s R

	N	Mean	95% CI	SD	Pearson’s R	*p*
Total Trauma Events	50	21	[16.1, 25.9]	17.2	0.44	0.001
Total Trauma Score	50	3.9	[3.3, 4.4]	1.9	0.26	0.06
Physical Abuse Events	50	8.1	[5.3, 10.9]	9.9	0.49	<0.001
Physical Abuse Events <18y	47	3.7	[2.2, 5.3]	5.2	0.44	0.002
Sexual Abuse Events	49	4.1	[2.1, 6.1]	6.9	0.16	0.29
Emotional Abuse/Neglect Events	50	6.7	[5.1, 8.4]	5.8	0.38	0.007

When looking at individual trauma types, women with higher counts of lifetime physical and emotional abuse events, as well as childhood physical abuse events, were found to experience increased loneliness (Table 3). Sexual abuse events were not associated with loneliness. To confirm the association between both physical and emotional abuse events and loneliness, the analyses were also conducted categorically with 3 groups of abuse events and remained statistically significant (Table 4).

**Table 4:** UCLA 3-Item Loneliness Scale by number of abuse events (categorical independent variables), ANOVA

	N	MLS	95% CI	SD	*p*
Physical Abuse Events <18yo					
0	27	4.5	[3.9, 5]	1.4	0.004
1-10	8	5.5	[4.4, 6.6]	1.3	
>10	12	6.4	[5, 7.8]	2.2	
Emotional Abuse or Neglect Events					
0	19	4.4	[3.8, 4.9]	1.2	0.029
1-10	5	4.6	[2.7, 6.5]	1.5	
>10	26	5.8	[4.9, 6.6]	2.1	

*MLS* Mean Loneliness Score

### Trauma narratives

Most (94%) of the women in this study shared backgrounds of trauma. Their experiences were vast, with frequent descriptions of repeat events or multiple types of trauma.

Many described physical abuse by caregivers and multiple participants reported severe abuse or being beaten to the point of significant physical injury, including broken bones. One woman shared:

*“My father used to punish us really strong... I guess that’s the way that he thought he would make us do right things. He used to, um, hit us with a wood, um, leather whip... they used to hit the horses with that. And he used to hit us so hard... the teacher one time, he told us to put our shirt up because he saw me with so many bruises, and he cried. He fell back and cried because my back was so bad.”*

Sexual abuse was also common, and participants frequently reported limited support surrounding disclosures—“*he was molesting me before I became a grown woman... my family told me to tell DHS I was mad at him and I’m lying.”* Some disclosures were met with outright denial:

*“One of the maintenance guys used to [abuse me]. I didn’t know what was going on at that age because I was too young. Then later on my aunt’s fiancé—wouldn’t no one ever believe me when I used to tell on him.”*

One woman reported molestation that knowingly occurred in exchange for the perpetrator financially supporting her family. Others believed their abuse to be a secret and never disclosed to the people in their life—*“I didn’t tell my mother, I didn’t tell my sister, I didn’t tell nobody.”*

Participants frequently reported histories of emotional abuse and neglect. One woman described the lasting ramifications of verbal abuse*—“in the back of mind I keep hearing him say, you know, ‘you ain’t shit. You not going to be shit.’”*

In addition to experiencing directed trauma, there were also numerous reports of witnessing violence or psychologically distressing events. Participants had witnessed domestic abuse, gun violence, and drug use—“*My stepfather... he used to chase my mom, my sister and, and myself out of the... apartment building where we used to live. Shooting. He was drunk and he just wanted to do that for the fun... and we were screaming and running.”*

It is important to recognize that the trauma experienced by this population was significant.

### Lifelong effects of trauma

Many women talked about the distress and shared how their traumas continued to affect them:

*“It still hurts because people who should have been there for me wasn't there... it's hard to find somebody to talk to.”*

*“I still feel that way... like, dirty. I feel like such a bad person.”*

*“You try your best not to remember and you wanna forget everything, but you can't. And I'm mad because I remember everything that happened to me in my past... I'm trying to push it out and I can't.”*

Women often described difficulties with trust and finding themselves in situations as adults that paralleled their previous experiences with abuse: *“everything that you do and you see as a kid... it does plays [sic] out sometimes... as you get [to] an adult, because you've seen it and you think you're accepting everything that happened then.”*

### Resilience

Despite this, they also displayed significant resilience, one woman sharing, *“I’m just trying to break that cycle because I’m not going to raise my kids like that.”* Others talked about the points in their lives when they were able to finally seek help*—“It wasn't until I turned 45 years old, that I started dealing with my issues on my own”—*or experiencing catharsis in sharing their stories or finding out they were not alone: *“I didn't wanna tell nobody that I was raped, and I heard... the girl in my class going through it, and I say, ‘This is a common occurrence. It's not an isolated event, you know?’”*

## Discussion

Trauma is highly prevalent and appears to be associated with increased loneliness in urban midlife economically-disadvantaged women. Though many studies have looked at the mental health ramifications of both trauma and loneliness separately, few have focused on the relationship they might share. Our study explores the experience of loneliness and the association with traumatic histories in midlife urban women, a predominantly low-income minority population.

Loneliness was common in this population, with 76% of women reporting some degree of loneliness. This rate is significantly higher than reports from general population surveys (22-35%) [14, 52]. In addition to high rates of loneliness, our study population also experienced high rates of trauma, with 94% having experienced potentially traumatic events. Though participants had elevated risk factors for trauma, including being mostly racial minority women from a low-income area, this does not appear to fully account for the extraordinarily high prevalence of traumatic histories. A recent primary care based study from Federally Qualified Health Centers examined trauma in socially disadvantaged and racial minority women—a population similar to ours [32]. Despite the similar racial and demographic make-up, women in the primary care study reported traumatic histories significantly less (65%) in comparison to our study population (94%). With the use of qualitative interviews, we also found that the trauma experienced by our study group would be considered severe.

Although it is unclear exactly what accounts for our population’s high rates of trauma, our urban setting, with high levels of community violence, may play a role. Additionally, our participants were selected from an ongoing longitudinal study and may have been comfortable disclosing traumatic events. Many had previously established relationships with study interviewers. To ensure our investigator-derived question regarding “living without a biological parent” (question 8) did not artificially skew results, the question was removed. The prevalence of trauma was then recalculated yielding the same results, with 94% of women (*N=*47) having experienced trauma(s).

Given that very few study participants reported no history of trauma (*N=*3), no direct comparison was made between the loneliness scores of these women and those who had reported historical trauma. Data from prior studies of US adults have already demonstrated an association between childhood and adulthood trauma and loneliness [28], the current study explored this connection further.

Associations were found between specific types of trauma and loneliness. Lifetime and childhood physical abuse, as well as emotional abuse/neglect, were associated with loneliness in adulthood. Though the population incongruity should be noted, a study of Israeli undergraduates found similar links, with associations between both childhood physical and emotional abuse and perceived social rejection [34]. They too found no link between sexual abuse and loneliness, though they only indirectly looked at loneliness (perceived social rejection) and reports of sexual abuse were low (4% of females), suggesting this type of abuse may have been underreported. Other studies have found contradictory results demonstrating a significant association between sexual abuse and loneliness [20]. Gibson and Hartshorne’s study looked specifically at childhood sexual abuse while our study looked at lifetime history, suggesting that childhood, and not adult sexual abuse/assault experiences, may contribute to loneliness later in life, though this requires further investigation.

In addition to trauma’s categorical association with loneliness, we also examined the cumulative relationship. Participants in the study were found to have experienced both numerous trauma events as well as multiple trauma types. The mean number of trauma events experienced by women was 21 while the mean for types of trauma experienced was 3.9. The current study demonstrates a positive correlation between the number of events and loneliness, suggesting that ongoing trauma or repeat victimization plays a greater role in loneliness symptoms than having experienced trauma in multiple domains.

Prior studies have found that both the cumulative number [55] of potentially traumatic events as well as types of abuse [8] have been associated with greater symptomatology in a variety of psychopathologies. Further, there is evidence that loneliness may mediate the relationship between trauma and adult psychopathologies [43]. Trauma, especially early repetitive abuse, has been associated with disturbances in self-organization characterized by negative self-concept, affect dysregulation, and subsequent difficulties with interpersonal relationships [29]. While the experience of trauma is often isolating in and of itself, it is also associated with feelings of detachment and emotional numbing. Some studies propose that early life trauma contributes to adult loneliness through impairments in attachment [36]. Feelings of shame, guilt, fear, and mistrust are frequently reported, and all were identified in relation to trauma by women in the current study. Often, women reported having never disclosed their trauma, having their experience denied/ignored, or feeling as though no one could understand what they were going through, all of which may contribute to ongoing feelings of loneliness.

### Strengths/limitations

Our results are strengthened by our ability to interview a primarily minority, low-income group of midlife women in their homes and community. Limitations of our study include a modest sample size and single study site. Our sample was a convenience sample from an existing community-based cohort, so participants may not be fully representative of women in the community. It is possible that women with a higher burden of mental health symptoms consented for inclusion in this study. Strong methods, including semi-structured interviews conducted within trusted professional relationships, provide for a greater appreciation and understanding of the lived experiences of midlife low-income women.

## Conclusion

The findings of this study highlight both trauma’s significant prevalence and relationship to elevated loneliness scores in urban midlife women. Loneliness was found to be significantly associated with lifetime and childhood physical abuse as well as emotional abuse/neglect. A positive correlation between cumulative trauma and loneliness also exists, with increasing numbers of traumatic events associated with greater loneliness scores.

Though further research is needed, the evidence supports screening for loneliness and trauma in the primary care and mental health settings of low-income communities with minority women. To most effectively and compassionately care for this population, trauma-informed care should be a mainstay of treatment. This includes creating a safe environment with educated staff and providers, screening for all individuals seeking care, and appropriate support and treatment, including referrals to external or community-based services. Given trauma’s association with and the effects of loneliness, an emphasis on fostering and supporting quality interpersonal relationships within the community is also vital. Beginning to mitigate the effects of trauma calls for addressing the trauma itself as well as the loneliness it may have fostered.

## Supplementary Information

**Additional file 1:** **Appendix A.** 3-item UCLA Loneliness Scale (UCLA-3) with revised scoring. **Appendix B.** Trauma Scale (TS).

## Data Availability

The data sets used in the current study may be made available by the principal investigator on reasonable request.
