# Supplementary material for: The unseen epidemic: trauma and loneliness in urban midlife women
Source: Womens Midlife Health. 2022 Oct 27;8:11. doi: 10.1186/s40695-022-00080-z (PMC9608918; doi:10.1186/s40695-022-00080-z)
Supplement: Supplementary file 1 — Additional file 1: Appendix A. 3-item UCLA Loneliness Scale (UCLA-3) with revised scoring. Appendix B. Trauma Scale (TS). [file 40695_2022_80_MOESM1_ESM.docx]

**Appendix A: 3-item UCLA Loneliness Scale (UCLA-3) with revised scoring**

1. How often do you feel that you lack companionship?

2. How often do you feel left out?

3. How often do you feel isolated?

| **Assessment scale/scoring** | **Revised scale/scoring** |
| --- | --- |
| Never (1) | Hardly ever (1) |
| Rarely (2) |  |
| Sometimes (3) | Some of the time (2) |
| Always (4) | Often (3) |

**Total Loneliness Scores range from 3 to 9**

**Appendix B: Trauma Scale (TS)**

In order to assess exposure to trauma in our study population, we developed a Trauma Scale (TS). Questions 1-7 were two-part questions (did a traumatic event occur, and if so, how many times). One point was given for every “yes” answer to a yes or no question and the total used to calculate a total trauma score. To calculate the total (number of) trauma events, responses to “how many times” were converted to a specific numerical response. Unanswered questions were scored as zero points.

| **Question** | 1. **Has this event ever happened to you?** | 1. **If so, how many times?** |
| --- | --- | --- |
| 1. Before age 18, were you ever physically punished or beaten by a parent, caretaker, or teacher? | Yes (1) No (0) | Once (1) 2-4 times (3) 5-10 times (7) > 10 times (12) |
| 1. Have you ever been in any situation in which you were seriously injured, or have you ever been in any other situation in which you feared you might be seriously injured or killed? | Yes (1) No (0) | Once (1) 2-4 times (3) 5-10 times (7) > 10 times (12) |
| 1. Has anyone, including family members or friends, ever attacked you with a gun, knife or some other weapon? | Yes (1) No (0) | Once (1) 2-4 times (3) 5-10 times (7) > 10 times (12) |
| 1. Have you ever seen someone seriously injured or killed? | Yes (1) No (0) | Once (1) 2-4 times (3) 5-10 times (7) > 10 times (12) |
| 1. Has anyone ever made you have intercourse or oral or anal sex against your will? | Yes (1) No (0) | Once (1) 2-4 times (3) 5-10 times (7) > 10 times (12) |
| 1. Has anyone ever touched private parts of your body, or made you touch theirs, under force or threat? | Yes (1) No (0) | Once (1) 2-4 times (3) 5-10 times (7) > 10 times (12) |
| 1. Have you ever been emotionally abused or neglected (for example, being frequently shamed, embarrassed, ignored, or repeatedly told that you were "no good")? | Yes (1) No (0) | Once (1) 2-4 times (3) 5-10 times (7) > 10 times (12) |
| 1. Were you ever put in foster care, put up for adoption, or live without at least one of your biological parents up to the age of 18? | Yes (1) No (0) | Once (1) 2-4 times (3) 5-10 times (7) > 10 times (12) |
| **Total Trauma Score** | **Total score**  (0-8) |  |
| **Total Trauma Events** |  | **Total score**  (0-96) |
